# Water, Sanitation, and Hygiene Risk Factors on the Prevalence of Diarrhea among Under-Five Children in the Rural Community of Dangila District, Northwest Ethiopia

**DOI:** 10.1155/2021/2688500

**Published:** 2021-08-21

**Authors:** Bizuayehu Hailu, Wu Ji-Guo, Tadesse Hailu

**Affiliations:** ^1^Department of Environmental Health, Guangdong Provincial Key Laboratory of Tropical Disease Research, School of Public Health, Southern Medical University, Guangzhou 510515, China; ^2^Department of Medical Laboratory Science, College of Medicine and Health Sciences, Bahir Dar University, Bahir Dar, Ethiopia

## Abstract

**Background:**

Under-five diarrhea is one of the major causes of morbidity and mortality in developing countries. Despite the tremendous achievement in reducing child mortality and morbidity in the last two decades, diarrhea is still the major causes of morbidity and mortality in resource-limited countries like Ethiopia due to the absence of clean water and poor sanitation and hygiene.

**Objective:**

This study aimed to assess the association of water, sanitation, and hygiene on the prevalence of diarrhea among under-five children in the rural community of Ethiopia.

**Methods:**

A cross-sectional study was conducted among randomly selected 419 under-five children from October to December 2021 in Dangila district, Northwest Ethiopia. A structured questionnaire was used to collect sociodemographic, environmental, and behavioral data. Data were entered into Epi Info and analyzed using SPSS software. Descriptive analysis was used to calculate the prevalence of diarrhea. Univariate and multivariate logistic regression were used to compute the association of water, sanitation, and hygiene with diarrhea. Statistical significance was considered if *P* < 0.05.

**Results:**

Among 419 participants, the prevalence of diarrhea was 106 (25.3%). The absence of handwashing habit of children (AOR = 7.70; 95% CI: 2.71–21.79) and caregivers after toilet (AOR = 19.10; 95% CI: 5.46–66.52), absence of latrine (AOR = 3.87; 95% CI: 1.24–12.08), playing with soil (AOR = 8.40; 95% CI: 4.58–36.66), and eating soil (AOR = 6.24; 95% CI: 1.99–19.78) were significantly associated with under-five diarrhea. Children who drink unprotected water were 2.21 times (AOR = 2.21; 95% CI: 0.51–9.69) more exposed to under-five diarrhea than who drink protected water, but it is not statistically significant (*P* = 0.29).

**Conclusion:**

The prevalence of under-five diarrhea is high in Dangila district. The absence of clean water and poor handwashing practice and the absence of latrine are the main factors associated with diarrhea. Therefore, strengthening water, sanitation, and hygiene strategy in the rural community should be prioritized.

## 1. Introduction

Diarrheal disease remains one of the major causes of mortality and morbidity among under-five children worldwide, especially in sub-Sahara African (SSA) countries which have poor sanitation and hygiene [1]. Diarrheal disease accounts for 1 in 9 child deaths worldwide and around 88% of the deaths are due to unsafe water, inadequate sanitation, and insufficient hygiene [2]. Diarrheal diseases are the third main cause of illness and death in under-five children in Africa and are accountable for an expected 30 million cases of severe diarrhea and 330,000 deaths in 2015 [3].

Under-five diarrhea is caused by protozoan parasites include *Giardia lamblia*, Cryptosporidium species, and *Entamoeba histolytica* [4], bacteria such as *Escherichia coli, Salmonella* spp*., Shigella* spp, and *Campylobacter jejuni*, and rotavirus among the viruses. Of which, rotavirus and *E. coli* accounted for the most common causes of diarrhea among under-five children [5, 6].

The factors that promote the existence of diarrheal diseases among under-five children are complex, and many of them are related to poor socioeconomic circumstances. The association of poor implementation of water, sanitation, and hygiene (WASH), unsafe human waste disposal, limited access to healthcare education, poor diet, and housing conditions in a community are driving force to under-five diarrhea [7]. These factors play a great role in the transmission of diarrhea-causing pathogen from an infected individual to the healthy one. Unless measure has been taken on the above listed factors, the burden of diarrhea will continue as a problem in a rural community where WASH is poorly practiced.

In Ethiopia, several strategies have been applied to decrease child mortality from under-five diarrhea. The Health Sector Development Program IV (2010/11–2014/15) in the country also aimed to reduce under-five mortality rate from 101/1000 live births to 68/1000. However, child morbidity and mortality in the country remains one of the highest in sub-Saharan Africa [8]. Some studies done in Ethiopia also show that under-five diarrhea is still a core public health problem that kills children, especially in the rural community [9, 10]. Although proper implementation of WASH reduces the occurrence of under-five diarrhea, it is poorly realized in the rural community of Ethiopia due to lack of awareness. Therefore, the aim of this study was to assess WASH risk factors on the prevalence of under-five diarrhea in Dangila district, Northwest Ethiopia.

## 2. Methods

### 2.1. Study Design, Area, and Period

A community-based cross-sectional study was conducted among under-five children in Dangila district, Awi zone, Amhara National Regional State, and Northwest Ethiopia from October to December 2020. Dangila is found in the north of Ethiopia (11°16′N 36°50′E) with an altitude of 2,137 meters above the sea level, 21°C mean annual temperature, and 1,260.7 millimeters mean annual rainfall.

All children aged 6 months to less than 5 years and lived in the study area for 3 months prior to data collection and their parents who were volunteered, gave consent, and filled the questionnaire during the data collection time were included in the study. Mothers/fathers of the selected children unable to respond to the questionnaire and critically ill during data collection were excluded from the study.

The sample size was calculated by single population proportion formula using 50% prevalence of diarrhea, 95% confidence level (*Z* = 1.96), and 5% margin of error (*d* = 0.05) because there is no previous study conducted in the study area [11].

(1) $$\begin{array}{c} n=\frac{Z_{a/2}^{2}\cdot P\left(1-P\right)}{d^{2}},\\ n=\frac{\left(1.96\right)2\left(0.50\right)\left(0.50\right)}{\left(0.05\right)2}=384. \end{array}$$

By considering a 10% (38) nonresponse rate, the final sample size was 422 (384 + 38).

### 2.2. Sampling Techniques

Among 30 kebeles, the smallest administrative units of districts in Dangila district, 15 kebeles (Abadira, Agaga, Chara, Dengeshita, Dimsa, Dube, Gisa, Gult, Gumdery, Manguda, Muksy, Quandisha, Quancha, Wufta, and Zelesa kebeles) were randomly selected and included in the study. Those households which had under-five children were screened and listed using a family folder at health posts based on the community-based information system. Then, households with under-five children were selected with a systematic random sampling technique. The number of under-five children in each Kebele was proportionally allocated from the total number of households which have under-five children in 15 selected kebeles. A systematic random sampling technique was used to select the households that have under-five children in each kebele. For those households with more than one under-five children, only one child was selected by the lottery method to be a part of the study.

### 2.3. Data Collection Techniques

#### 2.3.1. Questionnaire

A structured questionnaire was used to collect socio-demographic, health status of the under-five children and WASH-related data from mothers/fathers of under-five children through face-to-face interview and observational checklist. At the time of interview, children finger nails status (whether trimmed or not) and children shoes wearing status (whether wore or not) were checked. Questionnaires were filled by trained 4 nurses and supervised by two health officers.

### 2.4. Quality Control

Training was given for data collectors and supervisors prior to data collection. Pretest was conducted (in Wondefay kebele which is not included as a study kebele in Dangila district) to check whether respondents interpreting questions correctly and the order of the questions is not affecting the respondents answers by using 5% of the total sample sizes (nearly 22 participants) to standardize the questionnaires, data collection, and data processing. Identified problems during pretest were corrected before the start of actual data collection. Filled data questionnaires were cross-checked by the supervisors for their completeness, consistency, and accuracy. Generally, the reliability of data was assured during preanalytical, analytical, and postanalytical quality control steps.

### 2.5. Data Management and Analysis

Data were cleaned and entered into Epi Info version 6.04 and analyzed using Statistical Package for Social Sciences (SPSS) version 25 statistical software. The prevalence of diarrhea among under-five children was calculated by descriptive statistics. Univariate logistic regression was used to assess WASH-related factors associated with under-five children diarrhea. To resolve the confounding effect, variables with *P* < 0.25 in the univariate logistic regression were further analyzed by multivariate logistic regression analysis 95% confidence interval (CI). Variables with *P* < 0.05 in the multivariate analysis were considered as statistically significant.

### 2.6. Ethical Considerations

This study was ethically approved by Institutional Ethical Review Committee of Southern Medical University, School of international education, Department of Public Health. Supportive letter was obtained from Amhara Public Health Institute, Awi zone health office, and Dangila districts health office. Permission letters were secured from selected kebeles administrative offices. The purpose of the study was clearly explained to all parents of study participants before obtaining a written informed consent. All under-five children who had diarrhea during the data collection time were consulted to be taken to clinic and visited a doctor.

## 3. Results

### 3.1. Sociodemographic Characteristics of the Study Participants

From the total 422, 419 under-five children were participated in this study with 3 (0.7%) nonrespondent rates of their parents. The mean age of the study participants were 2.33 years, ranged from 0 to 4 years with standard deviation 1.11 years. The distribution of the study participants was higher (220 (52.5%)) in the age group of 0 to 2 years. The majority of children parents' educational statuses were able to read and write (202 (48.2%)), married (387 (92.4%)), and monthly income less than 11.33 USD (254 (60.6%)). The majority of under-5-year children (272 (64.9%)) were found in the family size of 5 to 9 (Table 1).

### 3.2. The Prevalence of Diarrhea in the Selected Kebeles

The total prevalence of diarrhea was 106 (25.3%; 95% CI: 22.2–29.8). Among the selected kebeles, the prevalence of diarrhea was higher (9 (29%)) in Dimsa followed by Agaga (8 (28.6%)) and Wufta (8 (28.6%)) kebeles. Lower prevalence rate of diarrhea was recorded in Zelesa (5 (18.5%)), Quancha (6 (21.3%)), and Dube (7 (22.8%)) kebeles (Table 2).

### 3.3. Diarrhea and Other Health Complications of Under-Five Children

The prevalence rates of diarrhea 56.6% (60/106) among females and 60.4% (64/106) in the age group of 0 to 2 years was higher than the respective males 43.4% (46/106) and 39.6% (42/106) in the age group of 3 to 4 years. The prevalence of loss of appetite among under-five children was 28.9% (121/419) which accounted for high prevalence (59.5% (72/121)) among females and (61.2% (74/121)) in the 0- to 2-year age groups. The prevalence of sense of tiredness among the study was (32.5% (136/419)) which was highly prevalent among female participants (52.9% (72/136)) and in the age group of 0 to 2 years (58.1% (79/136)). Under-five children who had vomiting accounted for (42.0% (176/419)). Of which, high vomiting was reported among males (52.8% (93/176)) and in the age group of 0 to 2 years (52.3% (92/176)). The prevalence of abdominal pain in under-5-year children was 32.2% (135/419) which accounted for (53.3% (72/135)) prevalence among females and (55.6% (75/135)) in the age group of 0 to 2 years (Table 3).

### 3.4. Prevalence of Diarrhea and WASH Indicators among Under-Five Children

High prevalence rate of diarrhea was obtained among under-five children who did not wash hands before their meal (49 (72.1%)), practiced open defecation (48 (33.8%)), drank river water sources (14 (53.3%)), and caregivers who did not wash hand before feeding under-five children (52 (76.5%)) (Table 4). Diarrhea was also high among under-five children who live in unclean environment (38 (84.4%)), a house with no handwashing facilities (90 (37.5%)), no solid waste disposal facilities (63 (53.3%)), and no liquid waste disposal facilities (80 (51.3%)) (Table 4).

### 3.5. Factors Associated with Under-Five Diarrhea

In the univariate logistic regression analysis, the presence of two under-five children per house, family size less than five, going to latrine with barefoot, playing with soil, eating soil, eating fruits and vegetable, having close contact with pets, absence of handwashing habit before meal and after toilet, having open defecation practice, no handwashing practice of caregivers, untrimmed nail status, drinking unprotected water source, and family monthly income less than 11.33 USD were associated (*P* < 0.01) with under-five diarrhea (Table 5).

In the multivariate logistic regression analysis, under-five children who play with soil were 8.40 times (AOR = 8.40; 95% CI: 4.58–36.66) more at risk than under-five children who did not play with soil. The habit of eating soil increases the odds of diarrheal infection by 6.28-fold (AOR = 6.24; 95% CI: 1.99–19.78) higher than under-five children who did not eat soil. Children who have close contact with pets were 4.73 times (AOR = 4.73; 95% CI: 1.78–12.59) more exposed to diarrhea disease than those who did not have close contact with pets. Under-five children who do not wash their hand after latrine were 7.70 times (AOR = 7.70; 95% CI: 2.71–21.79) more at risk than under-five children who washed their hand after toilet. The habit of caregiver not washing their hand before feeding their children increases the odds of under-five children diarrhea by 19.10-fold (AOR = 19.10; 95% CI: 5.46–66.52) higher than their counterparts. Under-five children who live in households which do not have latrine were 3.87 times (AOR = 3.87 : 95% CI: 1.24–12.08) more at risk than under-five children live a household which have latrine. Children whose parents have monthly income less than 11.33 USD were 2.90 times (AOR = 2.90; 95% CI: 1.04–8.08) more at risk to diarrhea than whose parents' monthly income is greater than 11.33 USD (Table 5).

## 4. Discussion

Diarrhea among under-five children is a common problem in developing countries, especially in the rural area, due to sanitation problem and predisposing environmental factors [12]. In this study, the total prevalence of diarrhea was 25.3% (95% CI: 22.2%–29.7%) which is higher than previous prevalence reports 10.77% in rural community of Enugu, South East Nigeria [13], and 22.1% in rural area of north Gondar, Ethiopia [12]. But it is lower than 29.9% in north central Ethiopia [14], and 32.6% in rural Burundi [15]. The difference might be due to the variation in the time period of data collection, socio-demographic characteristics of the study subjects, the sanitation and hygiene practice, the educational level of parents in the household level and study periods. The other justification might be only active cases of diarrhea were collected in this study.

In this study, higher prevalence of diarrhea (60.4%) was obtained in the age group of 0 to 2 years. This finding is consistent with earlier reports conducted in rural area of north Gondar [12]. The possible justification to lower age group might be the low level of immunity in lower ages of under-five children, high risk of eating contaminated foods and drinks, crawling starting time of kids, high contamination rate of hands of kids, and low sanitation practices.

The distribution of under-five diarrhea among households with no clean environment was 84.4% in this study. This result is supported by a previous report which shows children who live in a clean surrounding are less likely to be exposed to under-five diarrhea [16]. The possible justification might be open defecation is a common problem in rural area, and contaminated environment is a potential source of diarrhea-causing pathogens.

The absence of handwashing facility accounted for high prevalence of diarrhea (37.5%) in this study (*P* ≤ 0.001). This finding was supported by a previous report the presence of handwashing facility decreases the prevalence of under-five diarrhea in a rural community [12]. This could be justified as the knowledge of washing hands before eating in under-five children is low, especially in rural areas.

Good waste management system in a community helps to minimize diarrhea among children. The prevalence of diarrhea among households with no liquid waste disposal (51.3%) with (*P* ≤ 0.001) and no solid waste disposal (53.3%) with (*P* ≤ 0.001) were high in this study. This result is consistent with an earlier report that shows improper liquid and solid waste disposal is associated with under-five diarrhea [17]. This is possible that poor liquid and solid waste management can easily contaminate the soil where kids are playing.

In this study, under-five children having ingesting soil habit were significantly associated (*P*=0.002) with under-five children diarrhea. This result is consistent with previous reports [18, 19]. Playing with soil was also significantly associated (*P* ≤ 0.001) with under-five diarrhea, which is similar with previous study [20]. The possible explanation could be that soil is a potential source of pathogens that cause under-five children diarrhea, especially in rural area where eating soil and eating food with contaminated hands is a common phenomenon. Another explanation might be playing on the ground by making borehole—a type of game played by kids on the ground which contaminates the hands of children with soil which might contain pathogen.

Some diarrhea-causing pathogens can be transmitted by close contact with animals [21]. In this study, under-five children who have close contact with pets were significantly associated (*P*=0.002) with under-five children diarrhea. This result was similar to an earlier report [22]. The current report is also supported by previous report, close contact with domestic animals increases diarrhea among children [23]. This could be justified as zoonotic transmission of pathogens by close contact with domestic animals, including livestock, poultry, and companion animals, has been shown to play a role in the epidemiology and transmission of diarrhea-causing pathogens [21].

Good handwashing practices deceases the prevalence of diarrhea-causing pathogens. In this study, the habit of under-five children not to wash hands after toilet was significantly associated (*P* ≤ 0.001) with under-five diarrhea. Similar finding is obtained from a previous study [22]. In the same way, caregivers who do not wash hands before feeding their kids were also significantly associated (*P* ≤ 0.001) with under-five diarrhea, which is consistent with previous report [16]. This can be justified as children or their parents may go to latrine and feed without washing their hands which leads to the transmission of diarrhea-causing pathogens.

The absence of latrine in this study was significantly associated (*P*=0.020) with under-five diarrhea. This result is consistent with a previous report which shows that the availability of a latrine in the house decreases the prevalence of diarrhea [16]. This might be because improper utilization of latrine does not bury feces that contain diarrhea-causing pathogens.

Under-five children drinking unprotected (river and/or stream) water source were 2.21 times more at risk than those who drink protected (Tape and/or well) water source, but not statistically significant (*P*=0.290). This result is similar to previous study report [24]. This can be justified most water sources for animals and humans in the rural area are rivers which can be easily contaminated with human and animal excreta.

### 4.1. Limitation of the Study

Using a cross-sectional study design is one of the limitations for this study.

## 5. Conclusion

The prevalence of under-five diarrhea is high in Dangila district. WASH is also poorly implemented in the Dangila district. Poor handwashing habit of children and caregivers after toilet, absence of latrine, eating and play with soil, having close contact with pets, and family monthly income less than 11.33 USD are significantly associated with under-five diarrhea. Therefore, strengthening availability of clean water, effective sanitation, and hygiene strategy in the rural community should be prioritized to minimize the prevalence of under-five diarrhea. And also further studies with large sample size and study area should be done.

## Figures and Tables

**Table 1:** Sociodemographic characteristics of the under-five children and their parents in Dangila district, Northwest Ethiopia, 2021 (*N* = 419).

Variables		Number	Percentage
Age in years	0–2	220	52.5
	3-4	199	47.5

Sex	M	212	50.6
	F	207	49.4

Father educational status	Illiterate	163	38.9
	Read and write	202	48.2
	Primary	30	7.2
	Junior	19	4.5
	High school	0	0
	College	5	1.2

Mothers educational status	Illiterate	154	36.8
	Read and write	202	48.2
	Primary	32	7.6
	Junior	27	6.4
	High school	0	0.0
	College	4	1.0

Marital status of parents	Married	387	92.4
	Divorce	14	3.3
	Widowed	18	4.3

Monthly income in USD	<11.33	254	60.6
	11.33–22.66	142	33.9
	>22.66	23	5.5

Family size	2–4	147	35.1
	5–9	272	64.9

**Table 2:** The distribution of under-five children across their prevalence of diarrhea in the selected kebeles of Dangla district, Northwest Ethiopia, 2021 (*N* = 419).

Name of kebeles	Diarrhea status of under-five children		
	No, *n* (%)	Yes, *n* (%)	Total, *n* (%)
Abadira	24 (72.7)	9 (27.3)	33 (8.0)
Agaga	20 (71.4)	8 (28.6)	28 (6.7)
Chara	9 (75)	3 (25)	12 (3.0)
Dengeshita	22 (73.3)	8 (26.7)	30 (7.2)
Dimsa	22 (70.9)	9 (29)	31 (7.1)
Dube	24 (77.4)	7 (22.6)	31 (7.4)
Gisa	24 (72.7)	9 (27.3)	33 (7.9)
Gult	23 (74.2)	8 (25.8)	31 (7.4)
Gumdery	17 (77.3)	5 (22.7)	22 (5.3)
Manguda	23 (74.2)	8 (25.8)	31 (7.4)
Muksy	21 (75)	7 (25)	28 (6.7)
Quandisha	20 (76.9)	6 (23.1)	26 (6.2)
Quancha	22 (78.6)	6 (21.3)	28 (6.7)
Wufta	20 (71.4)	8 (28.6)	28 (6.7)
Zelesa	22 (81.5)	5 (18.5)	27 (6.4)
Total	313 (74.7)	106 (25.3)	419 (100)

**Table 3:** Prevalence of diarrhea and other health complications of under-five children across their age and sex in Dangila district, Northwest Ethiopia, 2021 (*N* = 419).

Variables of health complications	Sex (*n*)				Age in years (*n*)					
	M		F		0–2		3–5		Total	
	Yes	No	Yes	No	Yes	No	Yes	No	Yes	No
Diarrhea	46	166	60	147	64	156	42	157	106	313
Loss of appetite	49	163	72	135	74	146	47	152	121	298
Sense of tired	64	148	72	135	79	141	57	142	136	283
Vomiting	93	119	83	124	92	128	84	115	176	243
Abdominal pain	63	149	72	135	75	145	60	139	135	284

**Table 4:** Status of under-five diarrhea across WASH indicators among children in Dangila district, Northwest Ethiopia, 2021 (*N* = 419).

WASH indicators		Status of under-five diarrhea			*χ * ^2^, *P* value
		Yes, *n* (%)	No, *n* (%)	Total, *n* (%)	
Under-five children handwashing practice before their meal	Yes	57 (16.2)	294 (83.8)	351 (83.8)	93.92, ≤0.001
	No	49 (72.1)	19 (27.9)	68 (16.2)	

Open defecation practice of under-five children	No	58 (20.8)	221 (79.2)	279 (66.6)	8.99, 0.002
	Yes	48 (33.8)	92 (64.8)	142 (33.4)	

Caregiver hand wash practice before feeding their under-five children	Yes	54 (15.4)	297 (84.6)	351 (83.8)	112.48, ≤0.001
	No	52 (76.5)	16 (23.5)	68 (16.2)	

Drinking water source of under-five children	River	14 (53.3)	10 (41.7)	24 (5.7)	15.44, 0.001
	Stream	7 (25)	21 (75)	28 (6.7)	
	Well	32 (25.8)	92 (74.2)	124 (29.6)	
	Tape	53 (21.8)	190 (78.3)	243 (58)	

Presence of clean environment	Yes	68 (18.2)	306 (81.8)	374 (89.3)	93.32, ≤0.001
	No	38 (84.4)	7 (15.6)	45 (10.7)	

Handwashing facility	Present	16 (8.9)	163 (91.1)	179 (42.7)	44.26, ≤0.001
	Absent	90 (37.5)	150 (62.5)	240 (57.8)	

Solid waste disposal	Present	43 (13.8)	268 (86.2)	311 (74.2)	84.02, ≤0.001
	Absent	63 (53.3)	45 (41.2)	108 (25.8)	

Liquid waste disposal	Present	26 (9.9)	237 (90.1)	263 (62.8)	88.79, ≤0.001
	Absent	80 (51.3)	76 (48.7)	156 (37.2)	

**Table 5:** Bivariate and multivariate analysis of factors associated with diarrhea among under-five children in Dangila district, Northwest Ethiopia, 2021 (*N* = 419).

Variables		Diarrhea		COR (95% CI)	*P* value	AOR (95% CI)	*P* value
		Yes	No				
Number of <5 children/house	Two	40	21	8.43 (4.66–15.23)	≤0.001	1.62 (0.47–5.63)	0.447
	One	66	292	1			

Family size	≥5	21	31	2.25 (1.23–4.11)	0.009	2.45(0.74–8.39)	0.139
	<5	85	282	1			

Going to latrine with barefoot	Yes	51	41	6.15 (3.72–10.17)	≤0.001	2.45(0.84–7.13)	0.100
	No	55	272	1			

Playing with soil	Yes	82	86	9.02 (5.37–15.14)	≤0.001	8.40 (4.58–36.66)	≤0.001
	No	24	227	1			

Eating soil	Yes	51	30	8.75 (5.12–14.94)	≤0.001	6.28 (1.99–19.78)	0.002
	No	55	283	1			

Eating fruits and vegetable	Yes	35	43	3.10 (1.85–5.19)	≤0.001	1.99 (0.69–5.76)	0.207
	No	71	270	1			

Having close contact with pets	Yes	82	37	25.49 (14.42–45.06)	≤0.001	4.73 (1.78–12.59)	0.002
	No	24	276	1			

Hand wash habit before meal	Yes	92	298	1	0.005	1.59 (0.33–7.60)	0.559
	No	14	15	3.02 (1.41–6.50)			

Handwashing habit after toilet	Yes	116	241	1	≤0.001	7.70 (2.71–21.79)	≤0.001
	No	90	72	18.83 (10.40–34.08)			

Having open defecation practice of kids	Yes	48	92	1.99 (1.26–3.13)	0.003	1.06 (0.40–2.81)	0.903
	No	58	221	1			

Care giver handwashing habit	Yes	54	297	1	≤0.001	19.10 (5.46–66.52)	≤0.001
	No	52	16	17.88 (9.51–33.59)			

Kids nail status	Trimmed	69	268	1	0.001	2.76 (0.90–8.50)	0.076
	Untrimmed	37	45	4.49 (2.45–8.22)			

Presence of latrine	Yes	57	294	1	0.001	3.87 (1.24–12.08)	0.020
	No	49	19	13.03 (7.29–24.26)			

Drinking water source	Unprotected	21	31	2.25 (1.23–4.11)	0.009	2.21 (0.51–9.69)	0.290
	Protected	85	282	1			

Family monthly income in USD	<11.33	79	175	2.31 (1.41–3.77)	0.001	2.90 (1.04–8.08)	0.042
	≥11.33	27	138	1			

## Data Availability

Data are available in any open databases without limit including the Bahir Dar University website (https://bdu.edu.et/node/74).
